# Whole-Organ Isolation Approach as a Basis for Tissue-Specific Analyses in *Schistosoma mansoni*

**DOI:** 10.1371/journal.pntd.0002336

**Published:** 2013-07-25

**Authors:** Steffen Hahnel, Zhigang Lu, R. Alan Wilson, Christoph G. Grevelding, Thomas Quack

**Affiliations:** 1 Institute of Parasitology, Justus-Liebig-University Giessen, Giessen, Germany; 2 Centre for Immunology and Infection, Department of Biology, University of York, York, United Kingdom; McGill University, Canada

## Abstract

**Background:**

Schistosomiasis is one of the most important parasitic diseases worldwide, second only to malaria. Schistosomes exhibit an exceptional reproductive biology since the sexual maturation of the female, which includes the differentiation of the reproductive organs, is controlled by pairing. Pathogenicity originates from eggs, which cause severe inflammation in their hosts. Elucidation of processes contributing to female maturation is not only of interest to basic science but also considering novel concepts combating schistosomiasis.

**Methodology/Principal Findings:**

To get direct access to the reproductive organs, we established a novel protocol using a combined detergent/protease-treatment removing the tegument and the musculature of adult *Schistosoma mansoni*. All steps were monitored by scanning electron microscopy (SEM) and bright-field microscopy (BF). We focused on the gonads of adult schistosomes and demonstrated that isolated and purified testes and ovaries can be used for morphological and structural studies as well as sources for RNA and protein of sufficient amounts for subsequent analyses such as RT-PCR and immunoblotting. To this end, first exemplary evidence was obtained for tissue-specific transcription within the gonads (axonemal dynein intermediate chain gene SmAxDynIC; aquaporin gene SmAQP) as well as for post-transcriptional regulation (SmAQP).

**Conclusions/Significance:**

The presented method provides a new way of getting access to tissue-specific material of *S. mansoni*. With regard to many still unanswered questions of schistosome biology, such as elucidating the molecular processes involved in schistosome reproduction, this protocol provides opportunities for, e.g., sub-transcriptomics and sub-proteomics at the organ level. This will promote the characterisation of gene-expression profiles, or more specifically to complete knowledge of signalling pathways contributing to differentiation processes, so discovering involved molecules that may represent potential targets for novel intervention strategies. Furthermore, gonads and other tissues are a basis for cell isolation, opening new perspectives for establishing cell lines, one of the tools desperately needed in the post-genomic era.

## Author Summary

As a neglected disease, schistosomiasis is still an enormous problem in the tropics and subtropics. Since the 1980s, Praziquantel (PZQ) has been the drug of choice but can be anticipated to lose efficacy in the future due to emerging resistance. Alternative drugs or efficient vaccines are still lacking, strengthening the need for the discovery of novel strategies and targets for combating schistosomiasis. One avenue is to understand the unique reproductive biology of this trematode in more detail. Sexual maturation of the adult female depends on a constant pairing with the male. This is a crucial prerequisite for the differentiation of the female reproductive organs such as the vitellarium and ovary, and consequently for the production of mature eggs. These are needed for life-cycle maintenance, but they also cause pathogenesis. With respect to adult males, the production of mature sperm is essential for fertilisation and life-cycle progression. In our study we present a convenient and inexpensive method to isolate reproductive tissues from adult schistosomes in high amounts and purity, representing a source for gonad-specific RNA and protein, which will serve for future sub-transcriptome and -proteome studies helping to characterise genes, or to unravel differentiation programs in schistosome gonads. Beyond that, isolated organs may be useful for approaches to establish cell cultures, desperately needed in the post-genomic era.

## Introduction

Schistosomes are blood-dwelling digenean trematodes with a complex life-cycle comprising a freshwater intermediate snail host and a mammalian final host. Depending on the schistosome species, adults reside in the intestinal or urinary veins, predominantly of mammals [1]–[4]. Schistosome females produce hundreds of eggs per day, of which a significant proportion fails to pass into faeces (among others *Schistosoma mansoni*, *S. japonicum*) or urine (*S. haematobium*) to continue the life cycle but instead are dispersed by the blood stream into different organs where they can provoke severe inflammation, granuloma formation, hepatosplenomegaly, and even cancer [5]–[13]. Known as schistosomiasis (bilharzia) this infectious disease is considered by the World Health Organisation (WHO) as one of the most socioeconomically devastating parasitic disease worldwide, second only to malaria [14], [15].

Schistosomes are the only trematodes to have evolved separate sexes. Furthermore, a unique phenomenon of their biology is that females that have never been in contact with a male, are sexually immature and drastically smaller in size compared to paired, sexually mature females. A constant pairing contact to a male partner is the prerequisite for the differentiation of the female reproductive organs, which account for most of the significantly increased body size of a paired female [16]–[18]. As the eggs represent the causative pathogenic agents of schistosomiasis, the understanding of processes involved in reproductive organ differentiation, fertilisation, and egg-formation are of fundamental importance for understanding the reproductive biology of this exceptional parasite.

Praziquantel (PZQ) is the commonly applied drug combating all schistosome species in humans and animals, but is exclusively effective against adult worms [19]–[21]. Due to its use over decades there is an increasing fear of emerging PZQ resistance. Although cases of resistance have not been documented in the clinic yet, first evidence of reduced PZQ susceptibility in patients has been reported, and PZQ resistance can be generated *in vitro* [22]–[25]. This and the fact that no applicable vaccine is actually in sight emphasises the necessity for further research to find novel strategies combating schistosomiasis.

Considering the relevance of this parasitaemia, international genome sequencing projects have been completed with support of the WHO and other organisations [26]–[29]. As schistosome research has moved into the post-genomic era, numerous (sub-)transcriptome studies [30]–[45] as well as (sub-)proteome studies [46]–[53] have been initiated to functionally analyse tissue- and stage-specific gene transcription and expression to identify novel candidates for drug and vaccine development [33], [54]–[58]. These initiatives were paralleled by proteomics and glycomics to gain deeper insights in gene expression and regulation [59]–[62]. With respect to post-genomic studies, finally, the establishment of schistosomal cell lines has been recognized as a desirable tool. Although progress has been made during the last decades, the generation of permanently dividing cells has not been achieved yet [63]–[68].

In this study we present a novel, straightforward protocol for the isolation of pure and intact testes and ovaries from adult schistosomes. In contrast to other strategies requiring specific equipment and technical know-how to get access to different tissues [34], [41], [43] the introduced method is easy to handle, time saving and efficient by providing complete intact organs as well as tissue-specific RNA and protein of high quality and quantity for further analyses. As a proof-of-principle, the first molecular studies analysing the expression of candidate genes demonstrated the value of this approach for detailed characterisation of gene expression, which in one specific case (SmAQP) provided first evidence for tissue-specific, post-transcriptional regulation.

## Materials and Methods

### Ethics statement

All animal experiments have been performed in accordance with the European Convention for the Protection of Vertebrate Animals used for experimental and other scientific purposes (ETS No 123; revised Appendix A) and have been approved by the Regional Council (Regierungspraesidium) Giessen (V54-19 c 20/15 c GI 18/10).

### Parasite maintenance

A Liberian strain of *Schistosoma mansoni* was maintained in *Biomphalaria glabrata* as intermediate host and in Syrian hamsters (*Mesocricetus auratus*) as final host [69]. Adult worms were obtained by hepatoportal perfusion at day 42 post infection. Unisexual worm populations were generated by monomiracidial intermediate-host infection as described elsewhere [70]. Adult worms were transferred to Petri dishes of 60 mm diameter size containing 4 ml M199 medium (supplemented with 10% Newborn Calf Serum (NCS), 1% HEPES [1 M] and 1% ABAM-solution [10,000 units penicillin, 10 mg streptomycin and 25 µg amphotericin B per ml]) in groups of 20 couples, 25 males, or 50 females per Petri dish and kept *in vitro* at 37°C and 5% CO_2_. Immediately before processing, couples were separated by repeated pipetting and the use of featherweight tweezers.

### Isolation of testes and ovaries from adult schistosomes

Adult males or females (50–60 individuals each) were transferred separately into round-bottomed 2 ml-reaction vessels and washed twice with 2 ml of non-supplemented M199-medium at room temperature (RT). After removal of medium and addition of 500 µl of tegument solubilisation (TS)-solution (0.5 g Brij35 (Roth), 0.5 g Nonidet P40-Substrate (Fluka), 0.5 g Tween80 (Sigma), and 0.5 g TritonX-405 (Sigma) per 100 ml PBS (137 mM NaCl, 2.6 mM KCl, 10 mM Na_2_HPO_4_, 1.5 mM KH_2_PO_4_ in DEPC-H_2_O, pH 7.2–7.4)) the reaction vessels were incubated at 37°C and 1,200 rpm in a thermal shaker (TS-100, Biosan) for 5 min to solubilise the tegument in order to make the sub-tegumental musculature accessible for further processing. This step was repeated once (females) or twice (males) followed by three times washing with 2 ml of non-supplemented M199-medium at RT to remove most of the detergents.

Following removal of medium, the musculature consisting of outer circular muscles and inner longitudinal muscles was digested by protease treatment. To this end, elastase Type IV from porcine pancreas (Sigma, #E0258) was freshly dissolved in non-supplemented M199-medium to a final concentration of 5 units/ml and 500 µl added to each sample. Male- and female-containing reaction vessels were incubated with slight agitation (600 rpm) in a thermal shaker at 37°C for approximately 30–40 min, and the worms swirled up manually every 5 min. Progress of protein digestion was monitored by microscopic inspections of 20 µl aliquots. The appropriate time point to stop the reaction was achieved when the medium turned opaque and the female worms were fragmented, but not completely digested. At the same time the male worms appeared as a conglomerate of several flabby individuals. Additionally, some liberated reproductive organs were observed within these aliquots. Testes and ovaries were identified by their characteristic grape-like and peach-like shapes, respectively.

After addition of 1 ml non-supplemented M199-medium to each sample the whole content was decanted into Petri dishes of 60 mm diameter size. To completely harvest worm fragments/organs the vessels were rinsed three times with 1 ml of non-supplemented M199-medium. For quality inspection and following organ isolation, the digested worm batches were analysed under an inverted microscope. Most of the intact organs were liberated and ready to be harvested by pipetting. Remaining testes within worm-carcasses were set free by repeated pipetting (200 µl-tip). Ovaries surrounded by residual parts of the body wall were liberated in a similar fashion. For further purification of liberated testes and ovaries, the organs were collected with a 10 µl-pipette and transferred into 30 mm Petri dishes each containing 2 ml of non-supplemented M199-medium. If indicated, this step was repeated until the organs were completely free of any residual worm fragments or other cellular material. Finally, the organs were collected using a 10 µl-pipette and transferred into a 1.5 ml-vessel, and for concentration the testes and ovaries were centrifuged for 5 min at 1,000 g and 1 min at 8,000 g, respectively. The supernatant was carefully removed by pipetting and the organs frozen in liquid nitrogen before storage at −80°C for further RNA and protein isolation. With some practise the whole procedure takes approximately 1.5 hours. The use of freshly isolated adult worms is essential for the success of the described method as organ isolation failed with frozen worms. A schematic work flow is provided as supplementary Figure S1.

### Sample preparation and scanning electron microscope (SEM)-analyses

**Figure 1:**
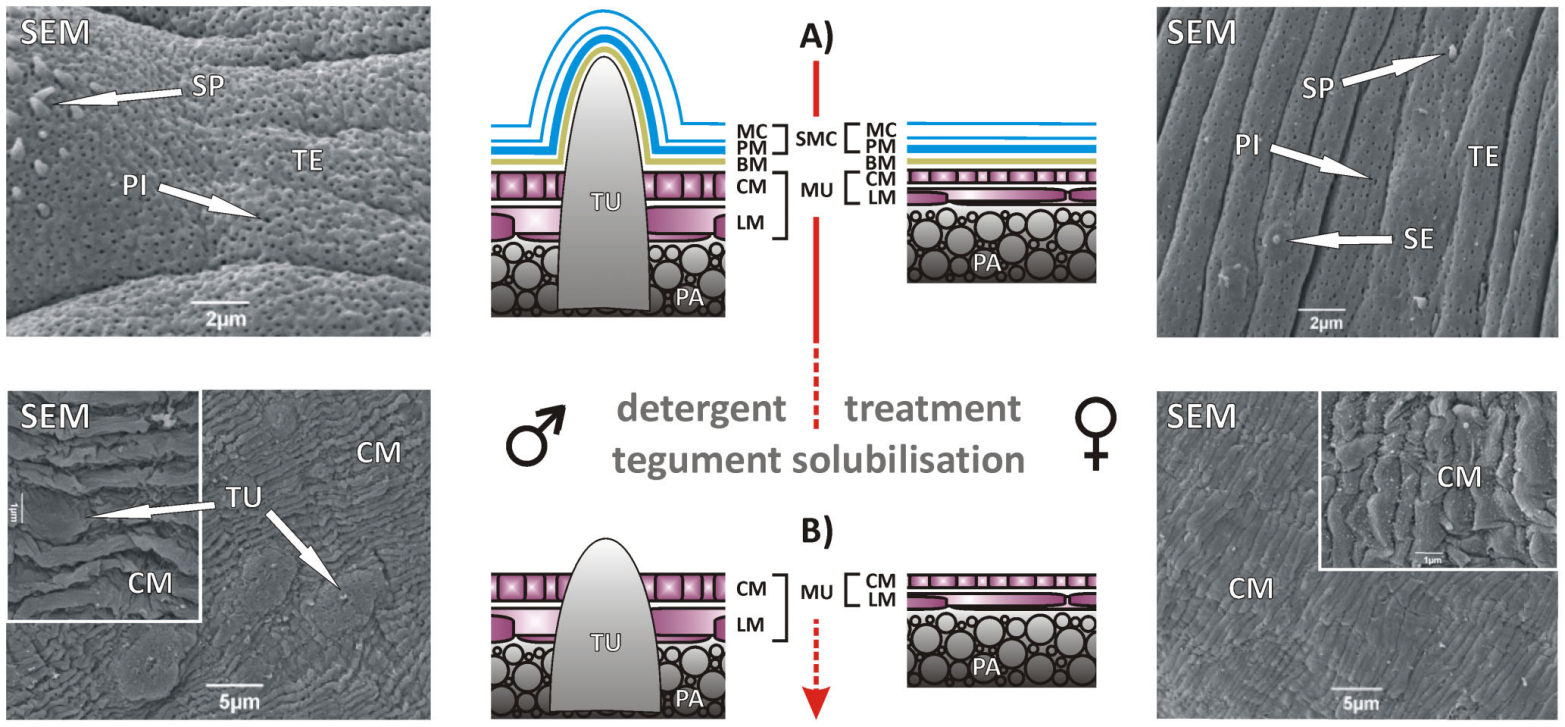
Schematic illustration and surface electron microscopy (SEM)-analyses of tegument solubilisation by TS-solution treatment. **A)** Untreated control males (upper left) and females (upper right) showing intact tegument (TE) with spines (SP), pits (PI), and sensory endings (SE). **B)** The tegument was completely removed due to detergent treatment exposing the outer circular muscles (CM) and the basis of the (male-specific) tubercles (TU). Membranocalyx (MC), plasma membrane (PM), longitudinal muscles (LM), basal membrane (BM), musculature (MU), parenchyma (PA); modified according to [107]; dashed arrow = continued in Figure 2.

Untreated adult control worms from *in vitro* culture and TS solution-treated worms with removed tegument (Figure 1) were washed three times with 2 ml of non-supplemented M199-medium and once with 2 ml PBS to remove Ca^2+^-ions. Subsequently, the worms were fixed in EM-fixative (2.5% glutaraldehyde, 4% formaldehyde in phosphate buffer (0.1 M final concentration, pH 7.2) over night (o/n), washed in several changes of buffer and then dehydrated through a graded series of increasing acetone concentrations to minimise shrinkage. They were critical-point dried, mounted on stubs and sputter coated with gold/palladium before viewing in a Jeol JSM-6490LV scanning electron microscope operating at 5 kV.

### Cell viability assay

Viability of gonad tissue-containing cells was analysed by Trypan Blue staining. To this end, freshly isolated testes and ovaries were carefully resuspended in 50 µl Trypan Blue solution (0.4% w/v, Sigma) in an Eppendorf-tube and incubated at RT for 5 min under slight agitation. The organs were sedimented by brief centrifugation at 1,000 g and 35 µl of the supernatant carefully removed by pipetting. After resuspension of the organs the residual 15 µl were transferred onto a microscope slide, covered with a cover slip and immediately analysed under the light microscope (CX21, Olympus). Images were acquired with a digital camera (SC30, Olympus) and analysed by CellSens Dimension software (Olympus).

### Isolation and analysis of total RNA from adult schistosomes and gonad tissue

Total RNA from adult schistosomes and gonad tissues was isolated using the PeqGOLD TriFast reagent (Peqlab) according to the manufacturers' protocol. In brief, five adult males and females as well as 50 testes and 50 ovaries were separately incubated with 500 µl TriFast-solution. The adult worms were mechanically homogenized with a plastic piston. After mixing with 100 µl chloroform and centrifugation for separating organic and aqueous phases, the upper aqueous supernatant, predominantly containing total RNA, was carefully removed to precipitate the RNA by adding 250 µl 2-propanol. In order to drive and visualise nucleic acid precipitation, 35 µg glycogen (RNase-free PeqGOLD glycogen, Peqlab) was added per 250 µl 2-propanol. Following incubation o/n at −20°C the RNA was concentrated by centrifugation (16,000 g), washed (by adding 500 µl 70% EtOH followed by another centrifugation step), and dried at RT. Finally, each RNA pellet was dissolved in 10 µl DEPC-H_2_O.

RNA quality and quantity were checked by electropherogram analysis employing the BioAnalyzer 2100 (Agilent Technologies). In brief, 1 µl of resuspended RNA was loaded on an Agilent RNA 6000 Nano Chip according to the manufacturers' instructions and analysed using the device setting “EukaryoteTotal RNA Nano assay”.

### cDNA-synthesis and RT-PCRs

Synthesis of cDNA was performed using the QuantiTect Reverse Transcription Kit (Qiagen) and 500 ng of total RNA per reaction. The preceding gDNA-wipeout step to eliminate residual gDNA as well as the following cDNA-synthesis using the RT-primer mix (included within the kit), which consisted of random hexamers and oligo dT-primers, were performed according to the manufactures' protocol. 1 µl of a 1∶40-dilution of each cDNA-sample was used in a standard PCR of 25 µl total volume (1× reaction buffer B: 80 mM Tris-HCl, 20 mM (NH_4_)_2_SO_4_, 0.02% w/v Tween20, 2.5 mM MgCl_2_, 200 µM dNTPs, 400 nM of each primer (Table 1) and 2.5 units Fire-Pol *taq* polymerase (Solis BioDyne)). PCRs were performed in a MasterCycler (Eppendorf) under the following conditions: 95°C for 2 min, followed by 35 cycles of 95°C for 30 sec, 60°C for 30 sec, 72°C for 30 sec and a final extension step of 72°C for 6 min.

**Table 1:** Target genes and RT-PCR primers.

Gene	Abbreviation	Accession-No. (NCBI)	Forward primer (5′→3′)	Reverse primer (5′→3′)	Ref.
*S. mansoni* Heat shock protein 70 (HSP70) gene	SmHSP70	L02415	TGGTACTCCTCAGATTGAGGT	ACCTTCTCCAACTCCTCCC	[109]
*S. mansoni* Immunophilin FK506 binding protein FKBP12	SmFKBP12	AY118110	ATGGGCGTTACCGTTGATACC	CCCAACCTCGAATTACTTTCCC	[75]
*S. mansoni* Calcineurin A	SmCNA	AJ276884	GTTTCTGGAACATGGACACCG	AGGGATCACTCGATGTGTTGG	[110]
*S. mansoni* TGFβ receptor kinase-1	SmTGFβR1	AF031557	AAACTCAGATCGTGTTGGAACC	AGCCGATTGACTAGCATACC	[111]
*S. mansoni* Aquaporin (AQP1)	SmAQP	EU780065	GACCAATCCGTCAGCATCTC	GATGAATAGGCCACCAACTTC	[74]
*S. mansoni* Axonemal dynein intermediate chain polypeptide, putative (Smp_167040)	SmAxDynIC	XM_002579327	TGGAGAAACGCAGGGAGATG	CAGCGAACTTCCCATACAGG	[29]
*S. mansoni* Permease 1 heavy chain	SmSPRM1hc	EF204542	CGAGTTTTACCCGTTTGATGAG	TTGACTACCAACTGGCTGATC	[72]
*S. mansoni* Nucleotide pyrophosphatase/phosphodiesterase 5 (NPP-5a)	SmNPP-5	EU570984	TGCTCCTAAGAAGTCAGCAGA	ATCTGTTGATATTGGCAAAGCTTC	[83]

### Extraction and analysis of total protein from adult schistosomes and gonad tissue

Adult schistosomes (50 males and 100 females), 280 testes, or 150 ovaries were separately washed once with 2 ml of non-supplemented M199-medium and PBS. 500 µl of 2× SDS sample buffer (200 mM Tris/HCl pH 6.8, 6% SDS, 10% β-mercaptoethanol, 20% glycerol, 20 mM pyrogallol, 1 tablet protease inhibitor cocktail (Roche)) and 100 µl of 1× SDS sample buffer were added to the adults and the reproductive organs, respectively. Worm samples were additionally sonicated 3–5 times with intermittent cooling until complete disruption. Samples were denatured at 100°C for 10 min and centrifuged for 10 min at 13,000 g. The supernatant was transferred to a fresh vessel and stored at −20°C. Protein samples from adults and gonads were diluted 1∶500 and 1∶250 in H_2_0, respectively. 2.5 µl of each dilution was used for protein concentration-determination by the BCA-method (Pierce) according to the manufacturers' instructions and re-analysed densitometrically on an SDS-PAGE by comparison to different amounts of a BSA-standard.

The quality of extracted proteins was analysed by 13% SDS-PAGE applying 1.2 µg proteins per lane followed by silver-staining. In brief, fixation of the gel was performed by slight agitation o/n in fixative (50% ethanol, 10% acetic acid, 0.0185% formaldehyde) and afterwards washed twice for 25 min with 50% ethanol. After sensitising with 0.02% Na_2_S_2_O_3_×5 H_2_O for one minute and washing three times with water, the gel was stained (0.2% AgNO_3_, 0.02775% formaldehyde) for 20 min. Subsequently, the gel was washed three times with water and transferred into another clean plastic bowl. Development was achieved by incubation in 6% Na_2_CO_3_/0.0004% Na_2_S_2_O_3_×5 H_2_O/0.0185% formaldehyde for 3 to 5 min until signals were clearly visible. Following brief washing with water the development was stopped by treatment in 12% acetic acid/44% ethanol for 10 min. Prior to storage in 1% acetic acid, the gel was washed three times with water for 10 min and scanned.

### Tegument protein extraction and precipitation

Pools of 100 males and 150 females were transferred into round-bottomed 2 ml-reaction vessels by pipetting and washed once with 2 ml of non-supplemented M199-medium and TS-solution at RT in order to remove most of the tegument-attached serum and host proteins. Subsequently, the worms were treated 6× with 500 µl TS-solution at 37°C and 1,200 rpm in a thermal shaker for 5 min to completely solubilise the tegument. The tegument protein-containing fractions were pooled (gender-separated) and precipitated by the chloroform/methanol method [71]. In brief, 1.4 ml of TS-solution supernatants were transferred into a 15 ml Corex-glass tube and mixed successively by vortexing with 5.6 ml methanol, 1.4 ml chloroform, and 4.2 ml H_2_O. After centrifugation for 10 min at 14,000 g, the upper aqueous phase was carefully removed, 4.2 ml methanol added to the bottom phase, vortexed, and centrifuged for 2 min at 14,000 g. Lastly, methanol was removed as much as possible without affecting the pellet, the precipitated proteins dried under vacuum, and finally resolved in 500 µl 2× SDS-sample buffer. Protein concentration was determined by the BCA-method as described before.

### Immunoblot-analyses

15 µg protein of each sample were separated by 13% SDS-PAGE and blotted onto a nitrocellulose membrane using a tank blot system (Roth). After washing the membrane with PBST (1× PBS containing 0.1% Tween-20), blocking was done with 1× RotiBlock (Roth) at RT for 30 min. The membrane was horizontally cut into four parts corresponding to the with respect to the size of the different target proteins. Subsequently, the strips were probed separately with the appropriate diluted rabbit-derived anti-sera [72]–[75]: SmSPRM1hc (Permease 1 heavy chain, 72 kDa, 1∶600), SmHSP70 (Heat shock protein 70, 70 kDa, 1∶20,000), SmAQP (Aquaporin, 33 kDa, 1∶600), and SmFKBP12 (FK506-binding protein, 12 kDa, 1∶3,000) o/n at 4°C. After washing three times with PBST for 15 min, the membranes were incubated with horseradish peroxidase (HRP)-conjugated goat anti-rabbit immunoglobulin G (IgG) diluted 1∶10,000 for one hour at RT. The strips were washed three times with PBST for 15 min and detection was performed by Enhanced Chemiluminescence (Pierce ECL Western Blotting Substrate, Thermo Scientific) and exposure to X-ray films (Kodak BioMax Light film).

## Results

### Isolation of testes and ovaries from adult schistosomes

In order to get access to different tissues, especially to the reproductive organs, a novel protocol was developed consisting of a combined detergent/protease treatment. Due to the morphological structure of trematodes, the first and crucial step is the removal of the surface membrane complex (subsequently referred to as tegument) appearing as a heptalaminate structure being composed of an outer trilaminate membrane forming the membranocalyx and a subjacent normal plasma membrane ([76]–[78], Figure 1A). The syncytial tegument of the adult schistosome worms is physiologically highly active and constitutes a strong and extremely resistant barrier against external influences. A combination of four different non-ionic and non-denaturing detergents (Brij35, Nonidet P40, Tween80, and TritonX-405) allowed the complete solubilisation of male and female teguments without destroying the integrity of the worm (Figure 1B). As confirmed by SEM-analyses, after tegument removal the musculature consisting of the outward circular muscles and the inward longitudinal muscles represented the outer surface of the remaining worm carcasses.

**Figure 2:**
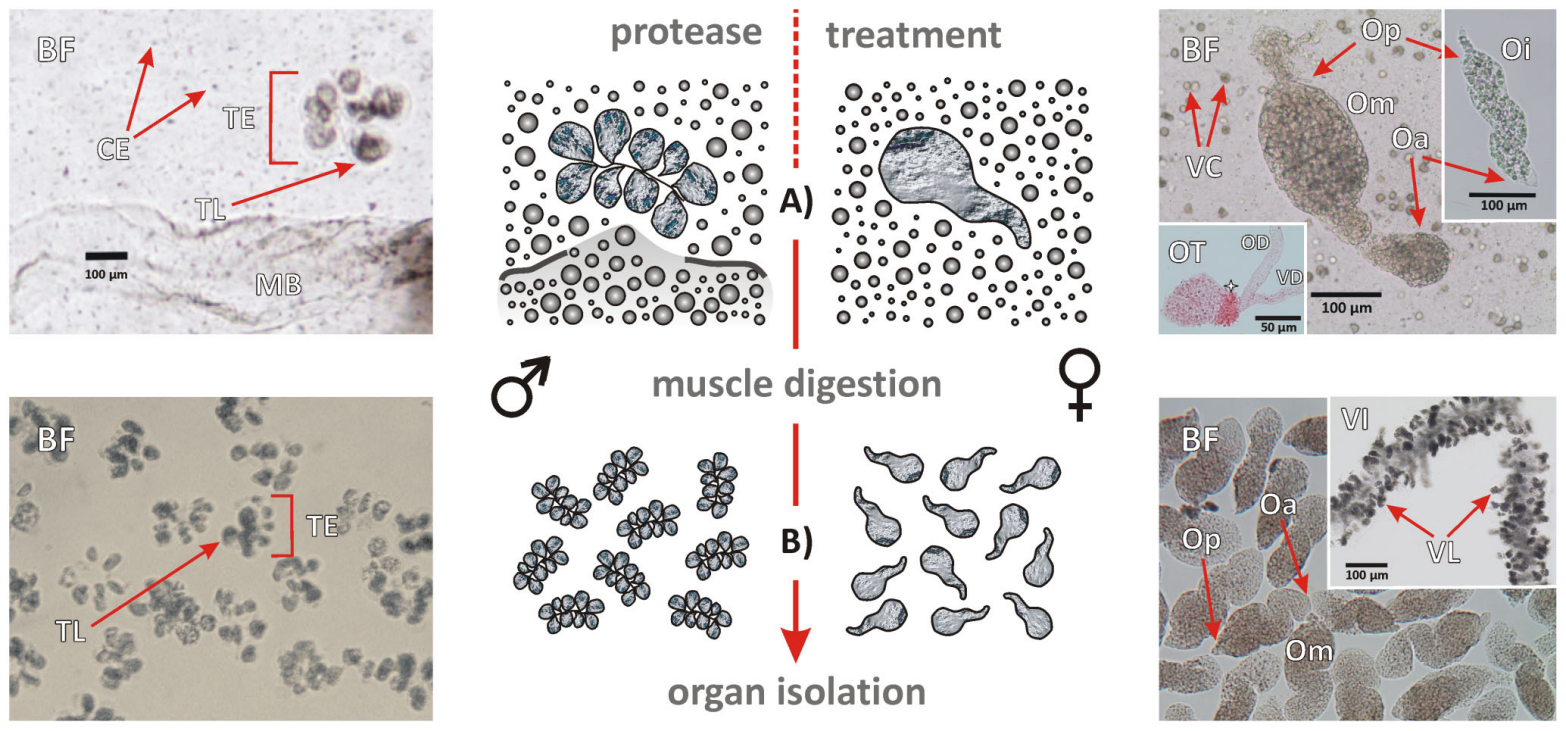
Schematic illustration and bright-field microscopy (BF) of gonad tissues following tegument solubilisation and protease treatment. **A)** Crude preparation of intact testes (TE) together with a part of an incompletely digested male worm body (MB) and different types of cells (CE) (left) and an mature ovary (Om) surrounded mainly by S4-vitelline cells (VC) from the vitellarium (right); immature ovary (Oi) and ootype (OT) with vitelloduct (VD) and oviduct (OD) isolated from a unisexual female; the ootype was contrasted by brief staining with Ponceau S; asterisk: “hymen”-like morphological structure typical for ootypes of unisexual females [18], [108] **B)** Mechanical transfer by pipetting led to the enrichment of pure testes (TE), mature ovaries (Om) after collecting and concentrating. TL (testes lobe), Op (ovary - posterior part containing mature primary oocytes in the case of mature ovaries), Oa (ovary - anterior part containing immature, stem cell-like oogonia); vitellarium (VI) with vitelline lobes (VL); dashed arrow = continued from Figure 1.

The proteinaceous musculature was carefully digested by elastase, which specifically hydrolyses elastin, a protein component of elastic fibres [79]. Digestion led to the degradation of the worm carcasses and thus to the release of intact reproductive organs and cells of different origin (Figure 2A). Testes and ovaries were easily identified by their characteristic grape-like and peach-like shapes, respectively, and further purified by sequential transfer (once up to several times) into new medium by careful pipetting (Figure 2B). The content of the testicular lobes appeared granular and homogenous, whereas the ovaries of mature females appeared in-homogenous as expected, containing immature oogonia in the small anterior and mature primary oocytes in the bigger posterior part, respectively [80]. Depending on the quality of preparation, the majority of testes were liberated consisting of 6–9 testicular lobes with a diameter of 90–100 µm per lobe (Figure 2A). With respect to females, over-digestion led to an increased fragmentation of the ovaries into posterior and anterior parts and, therefore, to a decreased yield of intact organs, whereas a low digestion-efficiency due to lower enzyme concentration/activity or time of digestion, led to higher numbers of ovaries surrounded by residual parts of the body wall. The size of isolated mature ovaries was approximately 400 µm in length and 120 µm in maximal width; as expected immature ovaries isolated from unisexual females were much smaller, about 200 µm length and 50 µm width. Average isolation efficiencies for gonads derived from bisexual infections were about 70%, whereas the efficiencies for testes and ovaries from unisexual infections were 70% and >90%, respectively. Finally, depending on further processing, the organs were either concentrated in reaction vessels by centrifugation, frozen in liquid nitrogen and stored at −80°C, or directly used for staining. Furthermore, it was even possible to isolate ootypes from unisexual females and vitellarium tissue from mature females (Figure 2A, B).

### Cell viability assay

**Figure 3:**
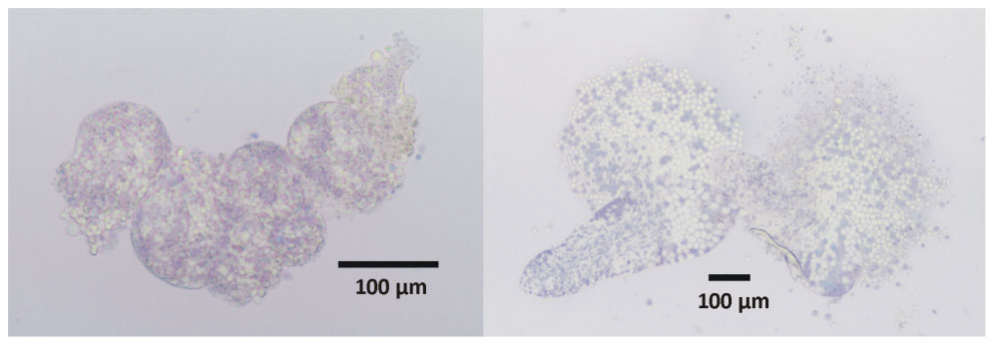
Vital staining of isolated reproductive organs. As an example one testis (left) and one ovary (right) derived from bisexual adult worms are shown, which were stained with 0.4% Trypan Blue immediately after isolation and examined by bright-field microscopy.

Freshly isolated testes and ovaries were stained with Trypan Blue to determine the viability of the cells within the isolated reproductive organs. This diazo dye penetrates cell membranes of dead cells exclusively but is not absorbed by living cells due to the selective exclusion by their intact cell membranes. Therefore, dead cells appear as blue-colour structures, whereas living cells appear more translucent, not being stained. The percentage of living cells within ovaries was estimated to be more than 60%, whereas with respect to testes the number of vital cells was found to be slightly lower and estimated by 40–50% (Figure 3). To determine if the quality of the isolated organs will be sufficient to serve as a source not only for viable cells but also for RNA and proteins, subsequent experiments were performed.

### Quantitative and qualitative analyses of isolated total RNA

**Figure 4:**
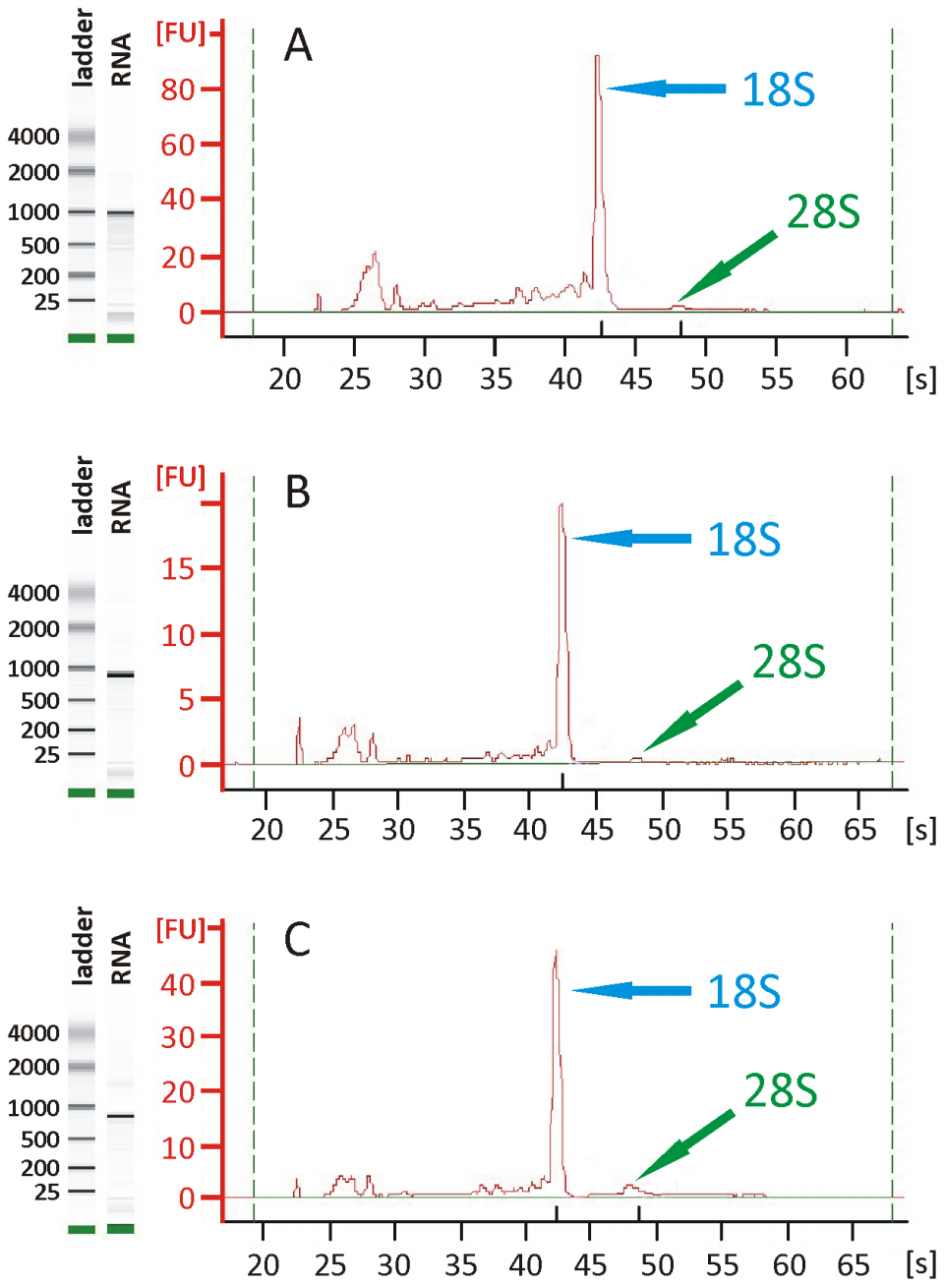
Quantitative and qualitative microfluid analysis of total RNA. RNA-analyses exemplarily shown for RNA isolated from adult males (**A**), testes (**B**), and ovaries (**C**) obtained by the organ isolation procedure were used. The figure shows a “gel-like image” consisting of the RNA-ladder and the appropriate total RNA sample (left) and the corresponding electropherogram (right); fluorescent units (FU), retention time (s).

Total RNA isolated from adult males, testes, and ovaries was analysed on an Agilent RNA 6000 Nano Chip (Agilent Technologies) for its integrity as this is an important prerequisite for further applications such as cDNA-synthesis and RT-PCRs. The analyses by this microfluidics-based system demonstrated that the quality of RNA isolated from reproductive organs was comparable to that obtained from adult male worms as control; no significant degradation of RNA was detected as proven by the integrity of the 18S rRNA shown by the appropriate peaks (Figure 4). Quantification of total RNA amounts of gonad tissue from bisexual as well as unisexual adults was done with the same system (Table 2). The average amount of total RNA per ovary derived from bisexual and unisexual individuals was determined to be approximately 26 ng and 0.8 ng, respectively. Independent of the pairing-status comparable RNA quantities of 8 ng and 7 ng per testis were determined. These differences were also reflected by the data obtained for whole adult worms of both gender and pairing-status as RNA amounts of males were comparable, whereas unisexual females yielded about 5-times less RNA compared to bisexual females. RNA content of bisexual males compared to females was similar.

**Table 2:** Quantification of total RNA and protein.

	Sample	Males		Females		Testes		Ovaries		mT
		bs	us	bs	us	bs	us	bs	us	bs
**Amount per individual/organ**	RNA [ng]	731	768	713	143	8.0	7.0	25.7	0.8	nd
	Protein [µg]	89.2	85.7	33.8	10.1	0.3	0.3 *	0.4	0.03 *	26.6
	RNA/Protein	8.2	9.0	21.1	14.2	26.7	23.3	64.3	26.7	**-**

The concentration of total RNA was determined with the BioAnalyzer 2100 (Agilent Technologies) employing an Agilent RNA 6000 Nano Chip using the device setting “EukaryoteTotal RNA Nano assay”. Protein concentration was determined by the BCA-method (Pierce) according to the manufacturers' instructions and re-analysed densitometrically on an SDS-PAGE by comparison to different amounts of a BSA-standard; nd = not determined; * = estimated by silver staining; bs, bisexual; us, unisexual; mT, male tegument.

### Gonad-specific RT-PCRs

**Figure 5:**
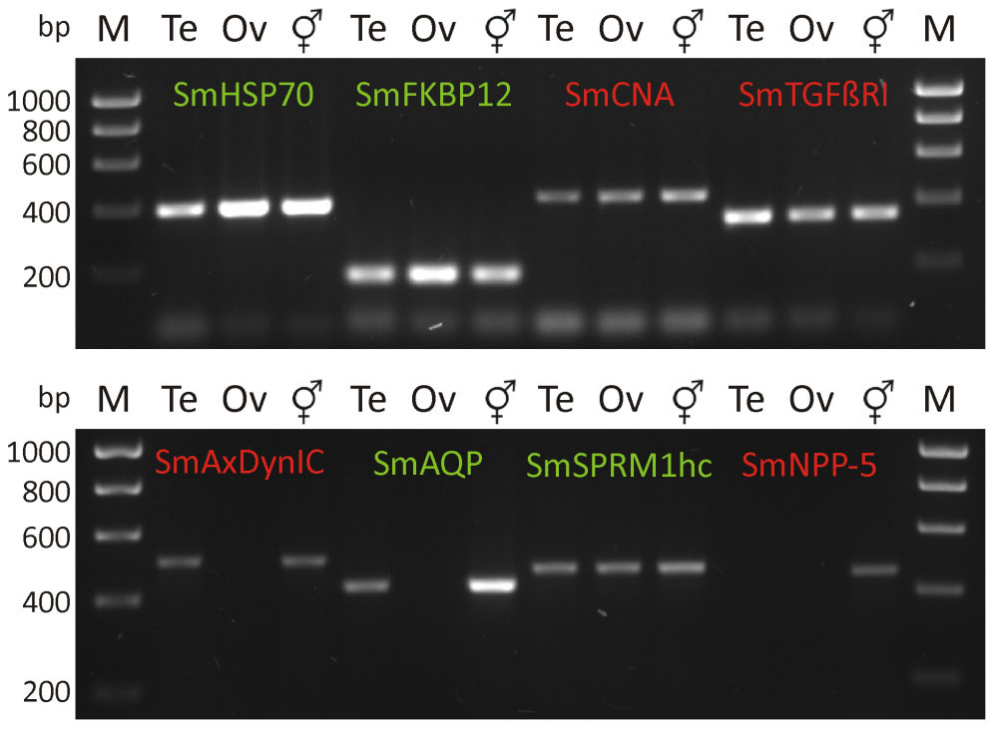
Gonad-RNA specific RT-PCRs. Total RNA of testes (Te), ovaries (Ov) and adult couples (merged Mars/Venus symbol) was isolated by Trizol and reverse transcribed. RT-PCRs were performed using gene-specific primers targeting SmHSP70 (Heat shock protein 70), SmFKBP12 (FK506-binding protein), SmCNA (Calcineurin subunit A), SmTGFβRI (Transforming growth factor β receptor I), SmAxDynIC (Axonemal dynein intermediate chain), SmAQP (Aquaporin), SmSPRM1hc (Permease 1 heavy chain), and SmNPP-5 (Nucleotide pyrophosphatase/phosphosdiesterase type 5); for references see Table 1. Marker (M) = Hyperladder I (Bioline). Target genes depicted in green were also analysed by immunoblotting (Figure 7).

Total RNA obtained from testes and ovaries as well as from adult couples was used for cDNA-synthesis and subsequent RT-PCRs. To demonstrate tissue-specific transcription, representative target genes (SmFKBP12, SmCNA, SmTGFβRI) were selected that had been reported in former studies to be transcribed and/or translated within testes and ovaries [81]. Further target genes (SmAQP, SmSPRM1hc, SmNPP-5) were shown to be preferentially, but not exclusively localised in the tegument of adult worms [82], [72], [83]. Additionally, SmHSP70 was chosen as it had been detected throughout diverse life stages and tissues as well as SmAxDynIC, which was expected to be expressed in testes due to its predicted function in sperm axonemes ([84], Table 1). As expected, most of the analysed target genes were transcribed in testes, ovaries, and adult couples, whereas the nucleotide pyrophosphatase/phosphodiesterase SmNPP-5 was not detected in the reproductive organs. Transcription of the amino acid transporter SmSPRM1hc gene was detected in the gonads of both genders providing indication for a function not restricted to the tegument. Interestingly, SmAQP was transcribed in testes but not in ovaries suggesting a role of this transporter for spermatogenesis but not for oogenesis. SmAxDynIC transcripts were found in testes but not in ovaries as anticipated (Figure 5).

### Quantitative and qualitative analysis of isolated total protein

**Figure 6:**
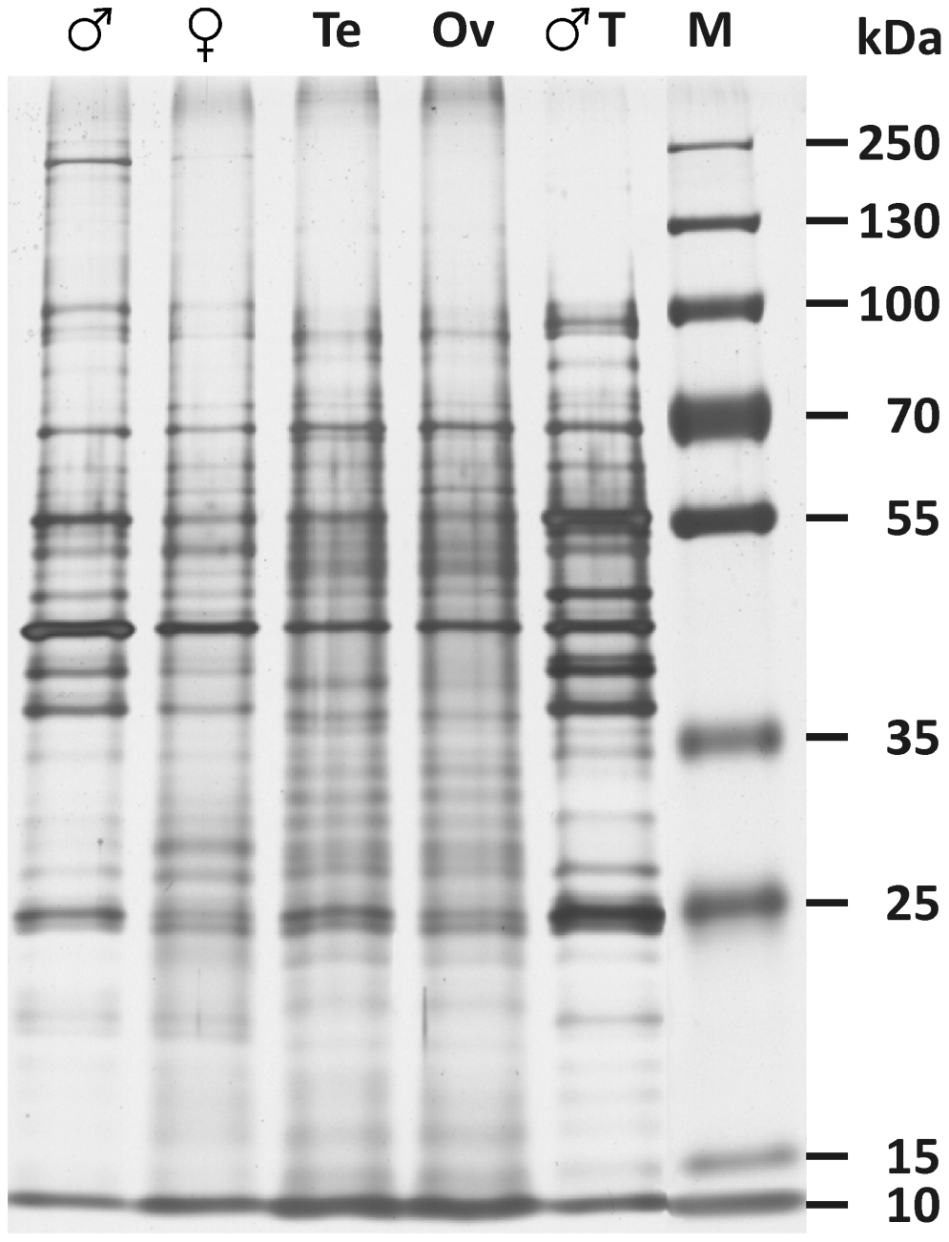
Protein patterns of *S. mansoni* organs/tissues and adults. 1.2 µg total protein from male (Mars symbol), female (Venus symbol), testes (Te), ovaries (Ov), and male tegument (T) were separated by 13% SDS-PAGE and visualised by silver staining. Marker (M) = PageRuler Plus Prestained Protein Ladder (Fermentas).

The quality of total protein was checked by SDS-PAGE analysis. To this end equal protein amounts derived from adult male and female, testes, ovaries, and precipitated male tegument fraction were separated on a 13% SDS-gel and subsequently silver-stained. All samples analysed showed a protein distribution over a wide molecular weight spectrum, ranging from 10 kDa to more than 250 kDa (Figure 6). Protein amounts of bisexual and unisexual adult worms as well as of the corresponding reproductive organs were determined by the BCA-method (Table 2). The average amount of total protein per ovary derived from bisexual and unisexual individuals was determined to be approximately 0.4 µg and 0.03 µg, respectively. Independent of the pairing-status comparable protein quantities of 0.33 µg (bisexual) and 0.3 µg (unisexual) per testis were determined. These differences were also reflected by the data obtained for adult worms of both gender and pairing-status as protein amounts of males were comparable, whereas unisexual females yielded 3.3-times less protein compared to bisexual females. Protein content of bisexual males compared to females was approximately 2.6-times higher. The proportion of male tegument protein based on the protein amount of one individual worm was about 27 µg. However, although a pre-washing step was performed it cannot be completely excluded that residual amounts of serum and/or host proteins were still present within this sample, and/or that additional proteins from inside the worms were co-extracted.

### Immunoblot-analyses on organ-specific protein

**Figure 7:**
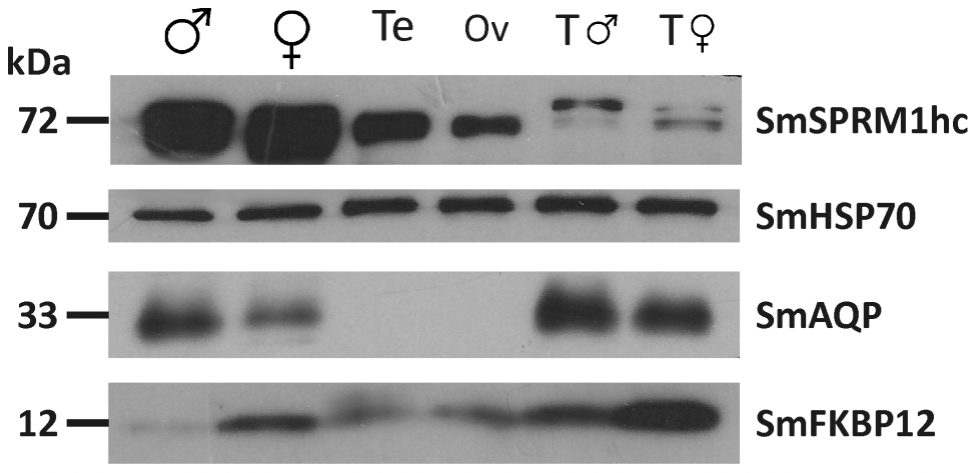
Gonad protein-specific immunoblots. 15 µg of total protein per lane isolated from adult worms (Mars and Venus symbol), testes (Te), ovaries (Ov), and tegumental proteins of both genders (T) were analysed by immunoblotting employing immune sera directed against SmSPRM1hc (Permease 1 heavy chain), SmHSP70 (Heat shock protein 70), SmAQP (Aquaporin), and SmFKBP12 (FK506-binding protein); for references see Table 1.

Tissue-specific protein expression of some of the genes previously analysed by RT-PCRs (Figure 5) was investigated also by immunoblotting employing antisera directed against SmSPRM1hc, SmHSP70, SmAQP, and SmFKBP12 (kindly provided by Patrick Skelly and Mo Klinkert). To this end equal amounts of protein derived from adult male and female, testes, ovaries as well as precipitated male and female tegument fractions were separated on a 13% SDS-gel and transferred on a membrane by electroblotting. Expression of SmSPRM1hc, SmHSP70, and SmFKBP12 was detected for all samples analysed, although only SmHSP70 showed a comparable strength of expression within every lane (Figure 7). Thus it served as a quantitative standard. Compared to this, SmFKB12 was more strongly expressed in females than in males. This was also observed for the appropriate tegument fractions, whereas the SmFKB12 expression levels within testes and ovaries seemed to be nearly identical. For SmSPRM1hc, the strongest signals were detected in adults of both genders. Weaker signals were detected for the reproductive tissue samples, and the weakest signals in the tegumental fractions. Whereas SmSPRM1hc seemed to be expressed slightly higher in testes and the male tegument compared to ovaries and the female tegument, it seemed to be slightly more highly expressed in adult females compared to adult males. SmAQP was found in adults and tegument samples of both genders showing minor dominance in male-derived samples, but it was absent from the gonad tissue.

## Discussion

In the light of published schistosome genome sequencing projects, methods to characterise genes of interest have gained importance in the dawn of the post-genomic era. Among other needs, the access to organs and cells for subsequent analyses is one of the desired aims [63]. First approaches towards cell isolation from schistosomes in the past were successfully performed by mincing adult worms under aseptic conditions and in the presence of trypsin/EDTA. This permitted the access to different kind of cell types suitable for cell culture purposes, but intact tissues and inner organs were disrupted due to mechanical forces [67], [68], [85]. Former attempts to isolate internal organs by a simple protease digestion were not successful (Grevelding, personal communication). Therefore, we established a novel method comprising a combined detergent/protease treatment. We assumed that the robust outer tegument had to be removed prior to digestion of the proteinaceous musculature by proteases. Indeed, solubilisation of the tegument turned out to be the crucial step as well as determining the appropriate detergents and their concentrations, as e.g. the use of SDS would lead to complete digestion. The combination of four different non-denaturing and non-ionic detergents was found to be optimal. A final concentration of 0.5% of each substance proved to be effective and the most gentle, completely solubilising the tegument but ensuring the integrity of the remaining worm body. Basically, the tegument could also be removed by a combined freezing/vortexing procedure [86], [87], but this will be lethal for schistosome cells. As for the detergents, the appropriate protease and concentration for muscle digestion had to be determined empirically. Initially, employing trypsin or chymotrypsin in the presence and absence of hyaluronidase or even proteinase K alone failed to digest the worms efficiently. Moreover, a collagenase/dispase-mixture dissolved in *Schistosoma* culture-medium [88] without addition of NCS appeared to be suitable for female, but not for male adult worms. Finally, elastase was found to be the enzyme of choice optimal for processing adult worms of both genders. However, this enzyme had to be dissolved in non-supplemented M199-medium. Applying the established protocol we succeeded to isolate intact testes and ovaries from adult males and females, respectively. After digestion, females appeared nearly completely fragmented resulting in liberated and intact ovaries as well as fragments, lobes, and huge numbers of released vitelline cells from the vitellaria, which represented mainly the S4-stage according to their yellowish appearance indicating a fully differentiated vitellocyte with lipids and yolk [89]–[91]. Using the same conditions, however, male worms were not digested completely, and appeared as agglomerated flabby carcasses. Nonetheless, testes were released easily by the procedure probably due to a dorsal vulnerability of the anterior male body, where the testes are located. The released reproductive organs were purified and enriched from the crude preparation by pipetting. This resulted in material free from other tissues or cells, which was essential for further subsequent experiments aiming to characterise tissue-specific gene expression. Average isolation efficiencies for testes and ovaries derived from bisexual as well as unisexual infections were shown to be sufficient to obtain enough material for subsequent experiments. Incidentally, also other organs such as intact ootypes from unisexual females as well as vitellarium tissue and gut fragments (data not shown) from bisexual females were obtained. Nonetheless, the presented protocol may serve as a basis for improvements to optimize the isolation of tissues other than gonads. We anticipate that the method is also applicable for other schistosome species as well as for further plathyhelminths having a tegument.

To assess the quality of the isolated reproductive organs and to judge if they could serve as a source for viable cells as well as intact RNA and proteins, testes and ovaries were stained with Trypan Blue immediately after isolation. Both types of organs contained numerous viable cells, which is a prerequisite for future attempts on establishing schistosomal cell lines. Future isolations of gonad tissues in the presence of anti-apoptotic substances/apoptosis-inhibitors will show if the amount of viable cells can be increased.

As a high percentage of cells within the isolated organs were proven to be viable, the question was addressed whether intact RNA could be isolated from this material. To this end total RNA extracted from reproductive organs of both genders and pairing-status as well as from corresponding adult worms were isolated and analysed by a microfluidics-based system. The results demonstrated that RNA isolated from reproductive organs was of comparable quality with respect to RNA obtained from adult males as shown by the integrity of the 18S rRNAs. The 28S rRNAs could hardly be detected, which is due to a small gap region within the 28S RNA molecule leading to the dissociation into two equal sized fragments [92], [93]. Nevertheless, isolation of reproductive organs from batches of 50–60 adult males or females will result in sufficient amounts of total RNA for further analyses like RT-PCRs and transcriptomics.

Quantity and quality of all protein fractions were verified by SDS-PAGE analysis confirming the absence of obvious protease-mediated protein degradation, as the proteins were spanning a broad molecular weight range. Accordingly, proteins of testes and ovaries can be isolated in sufficient amount and quality for further analyses such as immunoblotting and proteomics.

Comparison of bisexual males and females revealed 2.6-times more protein in males, whereas RNA amounts were similar between both genders. With respect to unisexual adult worms differences for RNA and protein amounts were determined as 5.4- and 8.5-times, respectively, which represents a 1.6-fold difference. These results indicate more post-transcriptional processes in bisexual compared to unisexual females. RNA amounts as well as protein quantities determined for testes derived from bisexual and unisexual males were comparable, which was anticipated with respect to similar sizes and morphology [80]. As expected, the differences regarding ovaries obtained from unisexual and bisexual females were significant. Ovaries derived from bisexual individuals were determined to contain 32-times and 14-times more RNA and protein, respectively, which is also explained by the influence of pairing on growth and differentiation of this organ in females. Deviations between RNA and protein amounts as reflected by the RNA/protein ratios are indicating a divergent transcriptional and translational activity with respect to these organs. Among others this can be explained by maternal transcription and storage of mRNAs [94] needed for subsequent embryogenesis and miracidial development within the egg. The RNA/protein ratio is generally considered to reflect the growth rate of cells indicating the level of synthesis activities. For example, this ratio was approximately 2.6-times higher in bisexual females than in bisexual or unisexual males indicating an increased RNA synthesis and cellular activity in paired, mature females. It was also observed that unisexual females, which are smaller having a much lower amount of total RNA and protein, have also a lower RNA/protein ratio, and thus probably less cellular activities. Whereas testes of bisexual and unisexual males have similar RNA and protein content, immature ovaries of unisexual females are smaller and contain lower amounts of total RNA and protein compared to mature ovaries present in bisexual females. Moreover, it is interesting to note that the RNA/protein ratio in ovaries of bisexual females is 3-times higher as in the corresponding complete worm, indicating a high synthesis activity within the ovary. In small ovaries isolated from unisexual females, this ratio is only 2-times more than in the complete organism, showing lower activity in the ovary of immature females.

To demonstrate the applicability of gonad-derived material for subsequent analyses, RT-PCRs and immunoblots were performed. To this end several target genes/proteins were examined that had been characterised in previous studies. SmHSP70 was detected with equal strength both on the transcription and expression level in the gonads of both genders and in all further samples analysed revealing the omnipresence of this molecule as shown in former studies [34], [35], [41], [47]–[49], [95], [96] as well as suggesting that HSP70 is ubiquitously expressed serving as a sample loading control for immunoblot-analyses.

SmFKBP12 and SmTGFβR1 as members of the TGFβ-signalling pathway have been shown before to be transcribed among others in the ovary but neither in the testis nor the sub-tegumental cells bodies of adult schistosomes by *in situ* hybridisation experiments [81]. With respect to the aforementioned study, SmFKBP12 expression was detected in the tegument of both genders, the ovary but not in testes by immunolocalisation. Contrary to these findings we demonstrated transcription and translation of SmFKBP12 as well as transcription of SmTGFβR1 also in the testes confirming TGFβ-signalling in the gonads of both genders. As FKBP12 has been reported to interact with the protein phosphatase calcineurin (CN), tissue distribution of the CN-subunit A (CNA) was also analysed previously by immunolocalisation [81] demonstrating SmCNA expression in the tegument and parenchyma of both genders as well as in the testes but not in the ovary. Again our own results show SmCNA-transcripts in the gonads of males and females. The partial discrepancies to the former results (summarised in Table 3) is explained by the detection limit of the *in situ*-hybridisation and immunolocalisation method as RT-PCR and immunoblot-analyses performed on the sub-transcriptomic and sub-proteomic level dramatically increases sensitivity regarding low abundantly occurring transcripts and proteins.

**Table 3:** Summary of gene-specific expression patterns.

Target	Method	Template	Testis	Ovary	Tegument	Ref.
**SmFKBP12**	*in situ*-hybridisation	5 µm-tissue sections	**−**	**+**	**−**	[81]
	immunolocalisation	5 µm-tissue sections	**−**	**(+)**	**+**	
	RT-PCR	organ-RNA	**+**	**+**	nd	*
	immunoblot	organ-protein	**+**	**+**	**+**	
**SmTGFβRI**	*in situ*-hybridisation	5 µm-tissue sections	**−**	**+**	**(−)**	[81]
	RT-PCR	organ-RNA	**+**	**+**	nd	*
**SmCNA**	immunolocalisation	5 µm-tissue sections	**+**	**−**	**+**	[81]
	RT-PCR	organ-RNA	**+**	**+**	nd	*
**SmNPP-5**	immunolocalisation	8 µm-tissue sections	nd	nd	**+**	[83]
	RT-PCR	organ-RNA	**−**	**−**	nd	*
**SmSPRM1hc**	immunolocalisation	7 µm-tissue sections	nd	nd	**+**	[72]
	RT-PCR	organ-RNA	**+**	**+**	nd	*
	immunoblot	organ-protein	**+**	**+**	**+**	
**SmAQP**	immunolocalisation	7 µm-tissue sections	nd	nd	**+**	[82]
	RT-PCR	organ-RNA	**+**	**−**	nd	*
	immunoblot	organ-protein	**−**	**−**	**+**	

* = current study, **+** = detected, **−** = not detected, **nd** = not determined.

Axonemal dynein intermediate chains have been described to function in cilia and flagella as well as in sperm axoneme assembly being important for spermatogenesis and fertility [84], [97]. Accordingly, SmAxDynIC transcripts were detected in testes but not in ovaries by organ-specific RT-PCRs and consequently could serve as a testes-specific marker.

Finally, three genes whose translation products had been shown before to be localised in the tegument of adult schistosomes were analysed as summarised in Table 3. SmNPP-5 transcription was neither detected in testes nor in ovaries by organ-specific RT-PCRs. This is consistent with previously published results showing the presences of SmNPP-5 predominantly in the tegument and at lower levels in internal tissues suggesting that SmNPP-5 is closely associated with the new tegument surface generation after cercarial penetration [83]. SmSPRM1hc is widely distributed throughout adult male and female worms as determined by immunolocalisation and is involved in the import of diverse amino acids [72]. Unfortunately, sections from adult couples analysed in the aforementioned study did not encompass reproductive organs. RT-PCRs performed on testes- and ovary-specific cDNA, however, revealed the transcription of SmSPRM1hc within both reproductive organs expanding knowledge about the distribution of this transporter and, furthermore, providing evidence for the importance of amino acid uptake also in the gonads. Consistently, SmSPRM1hc was detected by immunoblot-analyses to be expressed in the reproductive organs but also in the tegument of both genders. However, strongest expression was found in adult males and females indicating that SmSPRM1hc is widely expressed in many other tissues.

Aquaporins (AQPs) are small integral membrane proteins primarily involved in osmoregulation by transporting water across cell membranes. A subgroup of this protein family is additionally capable of transporting glycerol and therefore called aquaglyceroporins [98]. SmAQP, a type 3/9 aquaglyceroporin, was detected strongly in the tegument of both genders with slight predominance within the female fraction, which corresponded well with the results for whole control adult males and females. These results confirmed former findings that SmAQP is most strongly expressed in the tegument of 2-day and 7-day cultured schistosomula [74] as well as in the tegument of adult schistosomes where it was stronger for males than for females [82]. Furthermore, tegumental expression of a type 3/9 aquaglyceroporin was also supported by proteomic approaches [48], [49]. SmAQP was characterised to be capable of transporting water, mannitol, fructose, and alanine but not glucose, suggesting its important role in nutrient uptake and waste metabolite excretion [74], [82]. Immunolocalisation data with respect to the reproductive organs were missing in the aforementioned studies, and we showed that SmAQP translation products could neither be detected in testes nor in ovaries. In contrast, however, our RT-PCR results demonstrated the presence of SmAQP transcripts in testes but not in ovaries, which could be explained by post-transcriptional regulation and/or “leaky transcription”. Conclusively, our data emphasise former results [74], [82] that SmAQP is predominantly expressed and functional in the tegument but not in testes and ovaries. Nonetheless, indications for the existence and function of AQPs of both subgroups within the male reproductive systems in vertebrates but also in invertebrates have been obtained in the past. In rats, AQPs are present in germ cells as well as other tissues within the male reproductive tract and involved in the maturation of germ cells, the early stage of spermatogenesis, and in the cytoplasmic condensation occurring during differentiation of spermatids into spermatozoa [99]–[103]. AQPs were also identified in reproductive tissues of invertebrates indicating similar functions, as the expression of an aquaglyceroporin AQP3 homologue has been located in the seminal vesicle and vas deferens of *Caenorhabditis elegans* [104]. However, it cannot be excluded that AQPs of type1/2 exclusively transporting water might play a role in reproductive tissues of schistosomes, as AQPs homologous to AQP1 and AQP2 were shown to be expressed in the epithelial lining of ovary and testes in the trematode *Fasciola gigantica* [105].

Basically, tissue containing RNA and proteins can also be isolated by laser-assisted microdissection (LMD), which was previously shown to be a new method for tissue-specific profiling in schistosomes [34], [41], [43]. Downstream applications of LMD like RT-PCR, real time PCR, and microarray analyses are particularly suited because of the possibility of amplifying low amounts of extracted material. However, there is a potential risk of bias in such analyses, since low copy transcripts may not be detected in post-microdissection analyses. Furthermore, this method requires specific laboratory equipment, and the dissection has to be performed very precisely to prevent contamination with unwanted tissue material. Moreover, specimen preparation and preservation of the target material to prevent degradation during tissue processing is also challenging. Proteomic approaches are even more problematic as proteins cannot be amplified, which could be critical with respect to sensitivity. In the current study we present an alternative method for the isolation of schistosome tissues like reproductive organs as a source for RNA and proteins in sufficient amount, quality, and purity for further downstream analyses. The procedure is easy, inexpensive and quickly performed without the need of specific equipment. The amount of material obtained by this method helps to surmount detection limits of other methods like *in situ*-hybridisation or immunolocalisation as shown for SmFKBP12. Analyses on the basis of organ-specific cDNA can help to overcome such limitations and are useful for the validation of previously obtained results. Related to this, such sub-transcriptomic and sub-proteomic analyses are recommended to be performed for transcription and expression profiling of genes of interest prior to the performance of target-oriented experiments or prior to the postulation of working hypotheses. The developed protocol allowed also the isolation of very small tissues such as ovaries and ootypes of immature females, which are difficult to obtain by LMD due to the limited amount of accessible material. Future attempts in our group will concentrate on the enrichment of other tissues, e.g. vitelline lobes and the intestine by modifying the current protocol. Furthermore, the described technique opens new perspectives for the isolation of cells, which cannot be achieved by LMD. As cells with stem cell character will be among the isolated material [106] they represent an ideal source for new attempts to establish schistosomal cell lines. These could be of great value for e.g. a constant source of DNA and proteins of schistosomes, a system to express schistosome proteins in a homologous environment, for drug screening experiments and, if transfectable, gene characterisation.

## Supporting Information

Figure S1 Benchtop protocol depicting schematically the whole-organ isolation approach for the preparation of reproductive tissue from *Schistosoma mansoni* in a pure state. Arrow-headed semicircles with numbers = times of repetition; dashed arrow-headed semicircles = progressive digestion; dashed lines and arrows = appearance of treated worms at the corresponding step of the procedure.
(PDF)
