# Correction: Whole-Organ Isolation Approach as a Basis for Tissue-Specific Analyses in *Schistosoma mansoni*

**DOI:** 10.1371/annotation/7aad0212-557d-4121-a72f-da2c115ce534

**Published:** 2013-10-17

**Authors:** Steffen Hahnel, Zhigang Lu, R. Alan Wilson, Christoph G. Grevelding, Thomas Quack

There is an error in the composition of the TS-solution used for the solubilisation of the tegument contained in the Materials and Methods section. Instead of 0.5 g of each detergent, 0.1 g of each detergent was dissolved in 100 ml PBS.

The correct information is as follows:

After removal of medium and addition of 500 µl of tegument solubilisation (TS)-solution (0.1 g Brij35 (Roth), 0.1 g Nonidet P40-Substrate (Fluka), 0.1 g Tween80 (Sigma), and 0.1 g TritonX-405 (Sigma) per 100 ml PBS (137 mM NaCl, 2.6 mM KCl, 10 mM Na_2_HPO_4_, 1.5 mM KH_2_PO_4_ in DEPC-H_2_O, pH 7.2–7.4)) the reaction vessels were incubated at 37°C and 1,200 rpm in a thermal shaker (TS-100, Biosan) for 5 min to solubilise the tegument in order to make the sub-tegumental musculature accessible for further processing.

