# Supplementary figures and images for: Whole-Organ Isolation Approach as a Basis for Tissue-Specific Analyses in Schistosoma mansoni
Source: PLoS Negl Trop Dis. 2013 Jul 25;7(7):e2336. doi: 10.1371/journal.pntd.0002336 (PMC3723596; doi:10.1371/journal.pntd.0002336)

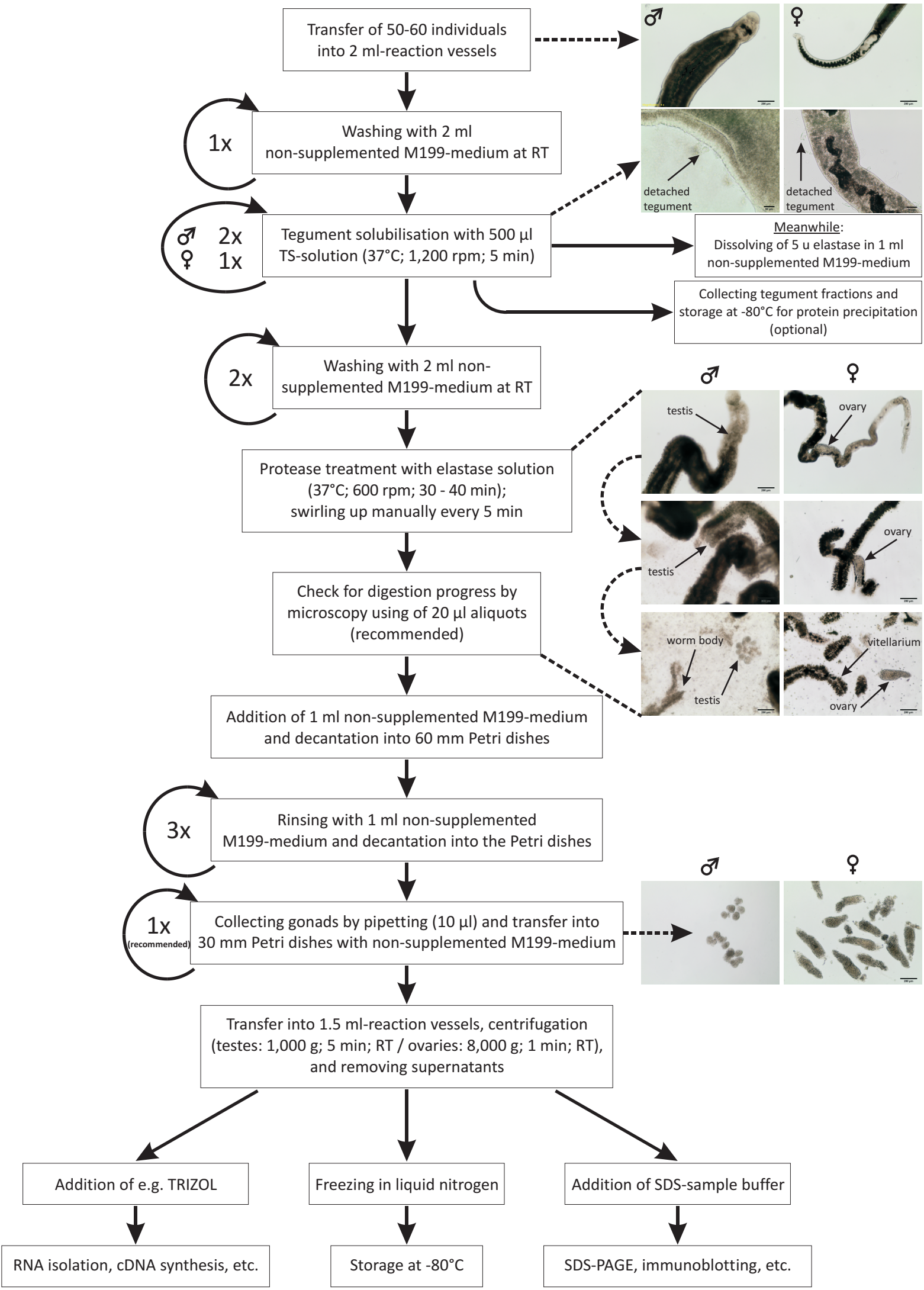

Supplement: Figure S1 — Benchtop protocol depicting schematically the whole-organ isolation approach for the preparation of reproductive tissue from Schistosoma mansoni in a pure state. Arrow-headed semicircles with numbers = times of repetition; dashed arrow-headed semicircles = progressive digestion; dashed lines and arrows = appearance of treated worms at the corresponding step of the procedure. (PDF) [file pntd.0002336.s001.pdf]
